# The value of magnetic resonance imaging to diagnose pathological complete response of rectal cancer after therapy: A protocol for meta-analysis

**DOI:** 10.1097/MD.0000000000012901

**Published:** 2018-10-26

**Authors:** Mei Zhang, Jipin Li, Xueni Ma, Bo Wang, Jiarui Wu, Ya Gao, Jinhui Tian, Jiancheng Wang

**Affiliations:** a Radiology Department, Gansu Provincial Cancer Hospital; b The Second Clinical Medical College of Lanzhou University; c Department of Nursing, Rehabilitation Center Hospital of Gansu Province, Lanzhou; d Department of Clinical Pharmacology of Traditional Chinese Medicine, School of Chinese Materia Medica, Beijing University of Chinese Medicine, Beijing; e Evidence-Based Medicine Center, School of Basic Medical Sciences, Lanzhou University; f Gansu Provincial Hospital, Lanzhou, China.

**Keywords:** diagnosis, magnetic resonance imaging, pathological complete response, rectal cancer

## Abstract

**Background:**

Although the trends of colorectal incidence rate and mortality have decreased during the past 20 years, however, they are still high. Neoadjuvant chemoradiotherapy is recommended as the standard treatment strategy of local advanced rectal cancer followed by surgery and adjuvant therapy. Predicting pathological complete response (pCR) accurately is relative to the next treatment strategy to avoid extensive therapy. And there are more and more physicians who would like to choose pelvic MRI imaging to evaluate the state of rectal cancer. Therefore, our analysis will aim to assess the value of MRI to predict pCR of rectal cancer after therapy and distinguish which sequence and magnetic strength is the best one to diagnose pCR.

**Methods:**

Comprehensive computer-based search will be performed using the PubMed, EMBASE, Cochrane Library, and CBM database (last updated in April 2018), 2 reviewers will extract the related information respectively. Pooled sensitivity, specificity, positive likelihood ratio, negative likelihood ratio, diagnostic odds ratio, and the area under the hierarchical summary receiver-operating characteristic curves will be calculated to estimate the diagnostic accuracy of different sequences and intensities of magnetic resonance imaging. Methodological quality will be assessed using the Quality Assessment of Diagnostic Accuracy Studies tool.

**Results:**

The results of this analysis will be submitted to a peer-reviewed journal for publication.

**Conclusion:**

The ability of different MRI sequences and magnetic intensities to identify pCR will be evaluated and the best one to diagnose pCR of rectal cancer after therapy will be recommended.

**Ethics and dissemination:**

Ethics approval and patient consent are not required, as this study is a meta-analysis based on published studies.

**PROSPERO registration number:**

CRD42018105672.

## Abstract 2

Supplemental Digital Content is available in the text

## 1 Introduction

Although the trends of colorectal incidence rate and mortality have decreased during the past 20 years, however, they are still high.^[1]^ Colorectal cancer is the third most common cancer in male and the second most common cancer in female around the world.^[2]^ Currently, the standard treatment strategy of local advanced rectal cancer is neoadjuvant chemoradiotherapy with 5-FU and 50.4 Gy followed by total mesorectal excision and adjuvant FOLFOX chemotherapy.^[3]^ However, there are some studies suggested that performing nonoperative therapy or conservative therapy to manage complete responders after neoadjuvant therapy for rectal cancer.^[4–6]^ Therefore, it is of high necessity to predict complete response accurately.

Pathological complete response (pCR) has been proposed as a surrogate endpoint for prediction of long-term clinical benefit.^[7,8]^ pCR is usually defined as no viable cells present on pathological examination of the surgical specimens, and it is relative to the next treatment strategy to avoid extensive therapy. Hence, predicting pCR after preoperative therapy accurately is necessary.

MRI performed at a higher field strength benefits from faster image acquisition, higher spatial resolution, and higher signal-to-noise ratio, which may improve the visibility of the rectal wall.^[9]^ And there are more and more physicians would like to choose pelvic MR imaging to evaluate the state of rectal cancer, because magnetic resonance imaging (MRI) has an important advantage in being able to evaluate iliac lymph nodes and can accurately assess the circumferential resection margin (CRM), which is key in planning surgery and in some countries informs the choice of neoadjuvant therapy.^[10]^ Therefore, among EUS, CT, MRI, PET, and PET/CT, we will mainly talk about MRI in our analysis.

There are studies have researched that MRI restaged TNM of tumor and predict circumferential resection margin after therapy.^[11,12]^ However, there is no study to evaluate the ability of MRI to predict pCR after therapy. This analysis will aim to assess the value of MRI to predict pCR of rectal cancer after therapy and distinguish which sequence and magnetic strength is the best one to diagnose pCR.

## 2 Methods

### 2.1 Design and registration

This systematic review and meta-analysis was registered with PROSPERO (registration number: CRD42018105672). We based the review methods on the Preferred Reporting Items for Systematic Reviews and Meta-Analysis Protocol (PRISMA-P)^[13]^ statement.

### 2.2 Information source and Search strategy

A computer-based systematic literature search will be performed using the PubMed, EMBASE, Cochrane Library, and Chinese Biomedicine Literature database from the earliest days until April 30, 2018. We will include studies which evaluated the diagnostic accuracy, sensitivity, specificity, positive predictive value (PPV), and negative predictive value (NPV) of rectal cancer used MRI. The search will be based on a combination of free text words and MeSH terms relating to rectal cancer, MRI, and diagnostic words, details on the search using the PubMed will be provided in Appendix.

### 2.3 Inclusion criteria

#### 2.3.1 Type of patients

Patients are confirmed with rectal cancer and have received neoadjuvant chemoradiotherapy will be included.

#### 2.3.2 Type of designs

Prospective or retrospective cross-sectional studies, case-control studies. and cohort studies will be included. Systematic reviews or meta-analyses will be also included to track their references.

#### 2.3.3 Type of interventions

MRI will be performed on each patient.

#### 2.3.4 Type of outcomes

The outcomes will be sensitivity, specificity, positive predictive value (PPV), negative predictive value (NPV) of MRI to predict pCR of rectal cancer. And then, true-positive (TP), false-negative (FN), false-positive (FP), and true-negative (TN) values will be calculated.

#### 2.3.5 Other criteria

There will be no limitations on language of publication, year of publication, and publication status. However, studies which have no sufficient data will be excluded.

### 2.4 Study selections

Literature search records will be imported into ENDNOTE X7 software. First, we will exclude duplicates. Then, 2 reviewers will select studies based on title and/or abstract and finally evaluate for inclusion based on the full text separately. Meanwhile, excluded studies and the reasons for their exclusion will be listed and examined by a third reviewer. Disagreements will be resolved by consensus.

### 2.5 Data extraction

A standard data extraction form will be created using Microsoft Excel 2013 (Microsoft, Redmond, WA, www.microsoft.com) to collect data of interest. For each included study, 1 reviewer will extract all relevant data using a standardized form, and the other reviewer will check these data. When the reviewers disagreed, they will discuss the data until consensus is reached. The extracted data will consist of first author, year of publication, country of the first author, language of publication, journal type, the influence factor of 2016 and funding; type of study, study design (prospective, retrospective, or unknown), period of study, location of study, patient enrollment (consecutive, random or not), the number of patients, the sex and age of patients, characteristics of disease and basic treatment of patients; the characteristics of index test and reference test, number of experts who assessed and interpreted the results of index test and reference test; the TP, FN, FP, and TN will be also extracted.

### 2.6 Quality assessment

We will assess the methodological quality of included studies using the Quality Assessment of Diagnostic Accuracy Studies (QUADAS-2) tool.^[14]^

### 2.7 Statistical analysis

For each study, we will construct the 2 × 2 table (with TP, FN, FP, and TN). According to different sequences and magnetic intensities, subgroup analysis will be performed. Thereafter, we will calculate the sensitivity, specificity, PLR, NLR, DOR, and HSROC. 95% confidence intervals (CI) for the summary estimates and likelihood ratios will be also calculated. Indirect comparisons of sequences and magnetic intensities will be performed respectively and potential publication bias will be investigated using Deeks funnel plot. We will use Stata SE 12.0 and MetaDiSc (version1.4) to perform the analyses. And we will plot the summary HSROC curves on a single, larger plot, with the curves superimposed using Review Manager 5.3. All studies will be presented as a circle and the summary point will be represented by a dot, which is surrounded by a 95% confidence region. The area under the HSROC curve will be calculated.

## 3 Discussion

In this meta-analysis, we will investigate the ability of MRI to predict pathologic complete response of rectal cancer after neoadjuvant chemoradiotherapy (CRT). Accurate prediction of pCR after neoadjuvant CRT can provide clinician treatment strategy. Maas et al^[4]^ suggested conservative policy for patients identified as complete responders after CRT. de Jong et al^[15]^ showed that the ability of MRI to exclude pCR is superior to its ability to confirm pCR.

In our study, we will perform standard pairwise meta-analysis and indirect comparison in different sequences and magnetic intensities. According to different sequences and magnetic intensities, subgroup analysis will be also performed. We hope that our analysis will identify the best sequence and magnetic intensity of MRI to diagnose pCR of rectal cancer after neoadjuvant CRT.

## Author contributions

JL, XM, JW and JT developed the study concept. MZ and YG developed the search strategy and selected studies. BW and JW extracted data and developed the statistical analysis and quality assessment. MZ and JL wrote the first draft of the protocol publication. JW reviewed the manuscript. All authors read and approved the final manuscript.

**Conceptualization:** Mei Zhang, Jipin Li, Jinhui Tian, Jiancheng Wang.

**Data curation:** Jipin Li, Xueni Ma, Jiarui Wu.

**Formal analysis:** Xueni Ma, Bo Wang.

**Methodology:** Ya Gao, Jiancheng Wang.

**Project administration:** Jiancheng Wang.

**Software:** Mei Zhang, Xueni Ma, Bo Wang, Jiancheng Wang.

**Validation:** Jiarui Wu.

**Writing – original draft:** Mei Zhang, Jipin Li.

**Writing – review & editing:** Mei Zhang, Jipin Li, Jiancheng Wang.

## Supplementary Material

Supplemental Digital Content
