# Supplementary material for: The value of magnetic resonance imaging to diagnose pathological complete response of rectal cancer after therapy: A protocol for meta-analysis
Source: Medicine (Baltimore). 2018 Oct 26;97(43):e12901. doi: 10.1097/MD.0000000000012901 (PMC6221727; doi:10.1097/MD.0000000000012901)
Supplement: Supplemental Digital Content [file medi-97-e12901-s001.docx]

**The value of MRI to diagnose pathological complete response of rectal cancer after therapy: a protocol for meta-analysis**

Mei Zhang, MD, Jipin Li, MD, Xueni Ma, MD, Bo Wang, MD, Jiarui Wu, MD, Ya Gao, MM, Jinhui Tian, PhD, Jiancheng Wang, MD*

**Appendix:**

**Search strategy using PubMed:** **#1** "Rectal Neoplasms"[Mesh] OR "rect* neoplasm*"[Title/Abstract] OR "rect* canc*"[Title/Abstract] OR "rect* carcinom*"[Title/Abstract] OR "rect* adenocarc*"[Title/Abstract] OR "rect* tumor*"[Title/Abstract] OR "rect* tumour*"[Title/Abstract] OR "rect* sarcom*"[Title/Abstract]

**#2** "Magnetic Resonance Imaging"[Mesh] OR nuclear magnetic resonance imaging[Title/Abstract] OR NMRI[Title/Abstract] OR NMR imaging[Title/Abstract] OR magnetic resonance imaging[Title/Abstract] OR MR tomography[Title/Abstract] OR MRI scans[Title/Abstract] OR MRI scan[Title/Abstract] OR MRI[Title/Abstract] OR functional MRI[Title/Abstract] OR functional MRIs[Title/Abstract] OR chemical shift imaging[Title/Abstract] OR magnetization transfer contrast imaging[Title/Abstract]

**#3** "Sensitivity AND Specificity"[Mesh] OR "False Positive Reactions"[Mesh] OR "False Negative Reactions"[Mesh] OR "ROC Curve"[Mesh] OR "Predictive Value of Tests"[Mesh] OR sensitivity[Title/Abstract] OR specificity[Title/Abstract] OR receiver operating characteristic[Title/Abstract] OR receiver operator characteristic[Title/Abstract] OR predictive value*[Title/Abstract] OR roc[Title/Abstract] OR pre-test odds[Title/Abstract] OR pretest odds[Title/Abstract] OR pre-test probability*[Title/Abstract] OR pretest probability*[Title/Abstract] OR post-test odds[Title/Abstract] OR posttest odds[Title/Abstract] OR post test probabilit*[Title/Abstract] OR posttest probabilit* [Title/Abstract] OR likelihood ratio*[Title/Abstract] OR positive predictive value*[Title/Abstract] OR negative predictive value*[Title/Abstract] OR false negative*[Title/Abstract] OR false positive*[Title Abstract] OR true negative [Title/Abstract] OR true positive*[Title/Abstract] OR fn[Title/Abstract] OR fp[Title/Abstract] OR tn[Title/Abstract] OR tp [Title/Abstract]

**#4 #1 AND #2 AND #3**
